# Multimodality approach to treat calciphylaxis in end-stage kidney disease patients

**DOI:** 10.1080/0886022X.2023.2256413

**Published:** 2023-09-19

**Authors:** Chloé Lajoie, Abdelaziz Ghanemi, Kateri Bourbeau, Aboubacar Sidibé, Yue-Pei Wang, Simon Desmeules, Fabrice Mac-Way

**Affiliations:** a CHU de Québec, L’Hôtel-Dieu de Québec Hospital, Faculty and Department of Pharmacy, Université Laval, Québec, Canada; b Department of Pharmacy, CSSS de la Minganie, Québec, Canada; c CHU de Québec Research Center, L’Hôtel-Dieu de Québec Hospital, Division of Nephrology, Faculty and Department of Medicine, Université Laval, Québec, Canada

**Keywords:** Calciphylaxis, calcific uremic arteriolopathy, multimodality approach, sodium thiosulfate, hemodialysis, chronic kidney disease, therapeutic response

## Abstract

A multimodality approach has been proposed as an effective treatment for calciphylaxis in patients with end-stage kidney disease. In this retrospective study, we report the cases of 12 end-stage kidney disease patients from l’Hôtel-Dieu de Québec hospital (Canada) who were diagnosed with calciphylaxis between 2004 and 2012 and treated with a multimodality clinical approach including sodium thiosulfate (STS). Statistical analyses were performed to evaluate the impacts of patients characteristics, the different interventions as well as therapy regimen on the therapeutic response. The majority of patients (*n* = 9) were hemodialyzed. The patients-associated comorbidities were consistent with previously reported risk factors for calciphylaxis: Diabetes (*n* = 11), calcium-based phosphate binders use (*n* = 10), warfarin use (*n* = 9), obesity (*n* = 7), female gender (*n* = 8) and intravenous iron use (*n* = 8). STS was given for a median duration of 81 days. 75% of the patients had a response (total or partial) including a complete response in 42% of patients. One-year mortality rate was low (25%). STS was used during a mean duration of 83.33 ± 41.52 days and with a total cumulating dose of 1129.00 ± 490.58 g. The recorded mean time before a complete response was 102.20 days (51–143). Pain improvement occurred after a mean time of 8.67 ± 10.06 days. None of the studied factors was statistically associated with a complete or a partial response to the multimodality approach. Although our data have a limited statistical power, they support treating calciphylaxis with a multimodality approach including STS as its effects are independent from important clinical variables.

## GRAPHICAL ABSTRACT

**Figure:**
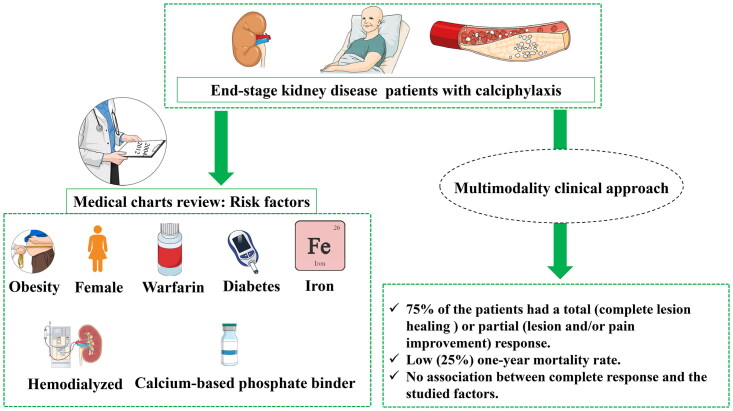

## 1. Introduction

Calciphylaxis or calcific uremic arteriolopathy [1] is a rare but highly morbid complication associated with chronic kidney disease (CKD) [2–4] that affects about 1% of hemodialysis (HD) patients [5–8]. It is characterized by medial calcification of cutaneous arterioles in the subcutaneous tissue resulting from calcium deposition in microvasculature [2,9]. Clinically, patients suffer from painful subcutaneous indurations that can progress into ulcerated lesions, tissue necrosis, infection, amputation and even death [5,10–13]; which makes calciphylaxis a life-threatening health problem [2]. It is a highly morbid condition, and one-year mortality has been reported to be as high as 70% in HD patients [5,10,14]. Risk factors that predispose to calciphylaxis in CKD have not been well studied, but long-term hemodialysis duration, female gender, obesity, diabetes and an elevated serum parathyroid hormone have all been pointed as risk factors [3,15]. Moreover, medications such as warfarin, activated vitamin D, calcium-based phosphate binders and intravenous iron have also been associated with the development of calciphylaxis in CKD population [16].

The rarity of calciphylaxis and the lack of studies regarding its treatment did not allow to validate an optimal therapeutic approach for calciphylaxis. The current recommendations are mostly based on opinions and it has been suggested for patients diagnosed with calciphylaxis to: 1) stop activated vitamin D and calcium-based phosphate binders, 2) lower dialysate calcium concentration, 3) increase dialysis frequency, 4) perform parathyroidectomy if needed, 5) use hyperbaric oxygenotherapy, and 6) in the recent years, to consider the use of bisphosphonates, cinacalcet, sodium thiosulfate (STS) [17–19]. In addition, SNF472 potential treatment, that is currently undergoing clinical trials and human amnion-derived mesenchymal stem cells (hAMSC) therapy, has shown promise as a regenerative treatment for severe calciphylaxis [20–24].

The exact anti-calcifying action of STS is currently unknown although multiple hypotheses have been previously suggested [25–28]. Furthermore, the factors associated with a therapeutic response to STS in calciphylaxis remain largely unknown. Therefore, reporting additional data on the treatment responses to STS - combined to other approaches - in patients with calciphylaxis represents a key step toward both validating STS as a therapeutic choice for calciphylaxis and optimize the adjunctive approaches. In this work, we report the results of a retrospective study on CKD patients suffering from calciphylaxis and treated with a multimodality approach including STS. We also describe determinants of the therapeutic success of our multimodal approach.

## 2. Study design and methodology

### 2.1. Selection of patients

**Figure 1.**
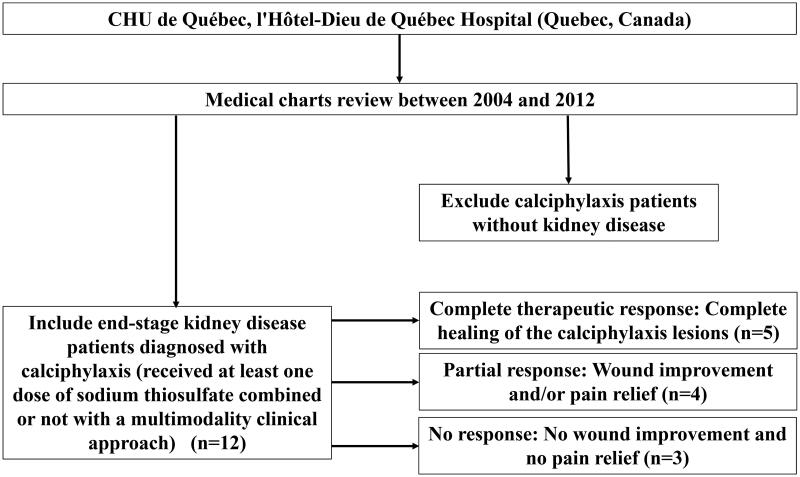
Cohort selection flow diagram.

This retrospective study has been conducted at CHU de Québec (l’Hôtel-Dieu de Québec Hospital), Québec, Canada. All stage 5 CKD patients diagnosed with calciphylaxis, thus received at least one dose of STS, between 2004 and 2012 were included in the study. Information about medical history, comorbidities, lesion evolution, diagnostic methods, laboratory analyses (Up to 12 months prior to diagnosis), pharmacotherapies and modifications in hemodialysis parameters were collected *via* medical charts review. Calciphylaxis patients without CKD were excluded. Thus, we included a total number of twelve patients as shown in Figure 1 (flow diagram). The study protocol, approved by the institution review board, was conducted in accordance with the Declaration of Helsinki.

### 2.2. Diagnosis and treatment of calciphylaxis

All patients diagnosis (either by nephrologists or dermatologists) was based on typical wound lesions. For 9 patients, calciphylaxis diagnosis was confirmed pathologically with a skin biopsy. For the remaining 3 patients, skin biopsies were not undertaken as clinicians judged that there would be an increased risk of wound complications and noninvasive tests were used to confirm the presence of calcification such as x-ray, bone scintigraphy which is a method used in calciphylaxis diagnosis (for the three patients who were not diagnosed with skin biopsies) [29–31] and mammography techniques. As the treatments of calciphylaxis evolved over the period of this study, the 12 patients did not receive exact similar therapeutic interventions. Our current clinical approach is based on a multimodal therapeutic approach that includes intensification of hemodialysis (frequency and/or duration), temporary decrease in calcium dialysate, cessation of vitamin D, calcium-based phosphate binders and warfarin, and STS with or without bisphosphonate or cinacalcet. The 12 patients of our study were also under medical supervision of chronic wounds care specialists who monitored the healing process throughout the study duration.

### 2.3. Therapeutic response

Lesions progression until healing was well documented in medical charts. Time to response was calculated from the date of the first STS dose until a complete or partial response. We also determined the STS time delay for each patient, which is defined by the time from calciphylaxis diagnosis until the date of the first STS dose. In total we had three possible therapeutic outcomes: Complete response, partial response and no response. The complete therapeutic response was defined as a complete healing of the calciphylaxis lesions, whereas partial response was defined as wound improvement and/or pain relief (from the medical chart review). For patients who had no wound improvement and no pain relief, this was marked as no response. Our definitions were based on previous literature and on the typical presentation of calciphylaxis that includes cutaneous lesions as well as pain [32–35]. A very recent paper also used similar (skin lesions changes and pain relief) approaches to evaluate therapeutic response to STS in calciphylaxis patients [36].

### 2.4. Potential determinants of therapeutic response

Various factors including age, sex, body mass index (BMI), biochemical analyses (from the patients medical records), calcium dialysate concentration change, dialysis frequency and duration and the post-diagnosis STS initiation delay were all considered as potential determinants of therapeutic response in calciphylaxis patients as suggested by previously published literature [10,37–39].

### 2.5. Statistical analysis

Statistical analyses were performed using SAS 9.4. Patients’ characteristics were described separately for each subject. Univariate analysis was performed to measure the association between potential determinants of the therapeutic response. Student T-tests were used to compare the means of continuous variables with normal distribution whereas Wilcoxon and Kruskal-Wallis Tests were used for the other continuous variables. The Fisher’s Exact Test was used to compare proportions for categorical variables. Spearman’s rank correlation test (GraphPad Prism 9.5.1) was used to assess the correlation of the total STS doses and the post-diagnosis delay before starting the treatment, the BMI, the age and the treatment duration for the patients with complete response. A p value inferior to 5% was considered as statistically significant.

## 3. Results and clinical data

### 3.1. Patients profiles and characteristics

Twelve patients with CKD stage 5 who were diagnosed with calciphylaxis were included in this study. Table 1 shows the demographic profile (age and gender), clinical data as well as the medications history of the patients at the time of the diagnosis. All patients were Caucasian with a mean age of 64.83 ± 9.99 years. They were mostly female (*n* = 8), obese (*n* = 7) and the main cause of end-stage of the CKD was diabetic nephropathy (*n* = 10). Nine patients were treated by hemodialysis for a mean duration of 58.67 ± 51.96 months. Three patients were not initially on dialysis but for two of them, hemodialysis was started once they were diagnosed with calciphylaxis. The majority of patients were on calcium-based phosphate binders (*n* = 10), warfarin (*n* = 9) and intravenous (IV) iron (*n* = 8). Eight patients had antibiotics (local application) for the treatment of wound infections.

**Table 1.** Patients characteristics and therapeutic profiles.

Pt	Age (Gender)	Dialysis vintage (months)	Lesion(s) site(s)	Diagnosis Method	Comorbidities	Intravenous Iron	Warfarin	Body mass Index
1	72 (F)	12	Abdomen	Skin biopsy	T2D, hypertension, CAD, PVD, CF, AF, DLP, gout	Yes	Yes	37.88
2	70 (M)	60	Abdomen, right leg	Skin biopsy	T2D, hypertension, CAD, primary hyperparathyroidism	Yes	No	44.75
3	64 (F)	None	Inferior limbs	Skin biopsy	T2D, heart transplant, DVT, episodic hypercalcemia, hypothyroidism, gout	No	Yes	19.84
4	70 (F)	12	Breast	Skin biopsy, mammography	T2D, AF, hypertension, PVD	Yes	Yes	37.46
5	48 (M)	156	Right calf	Skin biopsy	T2D, prior kidney transplant, MGUS-sarcoidosis, AF, PE, hypertension, DLP	Yes	Yes	26.48
6	48 (F)	84	Abdomen, thighs	Skin biopsy	T2D, CAD, PVD, hypertension, gout	No	No	29.40
7	68 (F)	None	Left thigh	Bone scan	T2D, hypertension, prior kidney transplant, CAD, DLP, IBS	No	No	45.45
8	76 (F)	48	Inferior limbs	Skin biopsy, bone scan	T2D, hypertension, CAD, AF, DLP, DISH	Yes	Yes	28.13
9	57 (M)	36	Inferior limbs	Bone scan	T2D, hypertension, CAD, DLP, hypothyroidism, osteoporosis	Yes	Yes	36.73
10	59 (M)	36	Right calf	Skin biopsy	T2D, hypertension, CAD, DLP, PVD	Yes	Yes	36.36
11	82 (F)	None	Inferior limbs	Skin biopsy, bone scan	hypertension, CAD, AF, DLP, COPD	No	Yes	19.69
12	64 (F)	84	Abdomen, thighs	Bone scan	T2D, hypertension, CAD, DLP, prior kidney transplant, hypothyroidism, gout	Yes	Yes	38.87

AF: atrial fibrillation; CAD: Coronary artery disease; CF: Cardiac failure; DISH: Diffuse idiopathic skeletal hyperostosis; DLP: Dyslipidemia; COPD: chronic obstructive pulmonary disease; DVT: Deep vein thrombosis; F: Female; IBS: Irritable bowel syndrome; M: Male; MGUS: Monoclonal gammopathy of undetermined significance; PE: Pulmonary embolism; Pt: Patient; PVD: Peripheral vascular disease; T2D: Type 2 diabetes.

### 3.2. Serum biochemical analyses at diagnosis

The serum calcium, phosphorus and alkaline phosphatase levels were all within normal ranges at the time of calciphylaxis diagnosis. The mean serum intact parathyroid hormone (iPTH) level was at 403.81 ± 244.07 pmol/L while only one patient had an iPTH level over 1000 pmol/L. The mean ferritin level was at 321.60 ± 217.89 μg/L. Glycemia was well controlled in diabetic patients (we had data for 8 among the 11 diabetes patients) as shown by the mean glycated hemoglobin level of 6.93 ± 1.20% (Table 2). In 6 patients where serum C reactive protein levels were available (data not shown), the mean value was slightly increased at 28.50 ± 16.31 mg/L (normal < 10 mg/L).

**Table 2.** Serum biochemical analyses at diagnosis.

Parameter	Mean ± standard deviation
Calcium (mmol/L)	2.15 ± 0.15
Phosphorus (mmol/L)	1.51 ± 0.3
Alkaline phosphatase (IU/L)	118.33 ± 74.78
Intact parathyroid hormone^a^ (pmol/L)	403.81 ± 244.07
Albumin (g/L)	35.09 ± 3.92
Ferritin (μg/L)	321.60 ± 217.89
Glycated hemoglobin (HbA1c), %	6.93 ± 1.20

^a^ The value for each patient represents the average of measures conducted during the 12 months prior to the calciphylaxis diagnosis (1–5 measures for each patient) *n* = 8–12 patients.

### 3.3. Characteristics and diagnosis of calciphylaxis

Calciphylaxis lesions at diagnosis were characterized by the presence of wounds, subcutaneous indurations and pain. The majority of the lesions were located on the inferior limbs (*n* = 10) including three patients who had concomitant lesions on the abdomen (Table 1). One patient had a single lesion on the breast. The diagnosis was made clinically and then confirmed by a skin biopsy of the lesion (*n* = 10) among which we had skin biopsy in addition to an imaging procedure (*n* = 4) or only with an imaging procedure alone (*n* = 2). Lesions biopsies were not performed in every patient either because the lesions appearance was considered to be typical or there were concerns about worsening the preexisting lesions. Imaging procedures were performed by bone scintigraphy in five patients and mammography in one patient.

### 3.4. Sodium thiosulfate treatment

STS was typically given IV three times per week at a dose of 25 g given during the last hour of the dialysis session. STS dose and/or frequency would be reduced afterwards when patients developed side effects. We stopped STS in three patients who had major side effects. The mean time delay before starting STS treatment following initial calciphylaxis suspicion was 55.83 ± 75.75 days. A complete response occurred in five (42%) patients, STS was used during a mean duration of 83.33 ± 41.52 days and with a total cumulating dose of 1129.00 ± 490.58 g (Table 3) which corresponds to a median of 1095 g and an interquartile range (IQR) of 1075. The recorded mean time before a complete response was 102.20 days (51–143). Overall, 75% of the patients had either a partial or complete response. Pain improvement occurred after a mean time of 8.67 ± 10.06 days. On univariate analysis, no specific factor (patient characteristics, comorbidities, lesion site, biochemical parameters, treatment modalities, etc.) was found to be associated with complete (Table 4) or partial response (data not shown). Three patients died within the year following calciphylaxis diagnosis.

**Table 3.** Clinical responses to sodium thiosulfate, side effects and multimodal interventions.

**Patient**	**Complete response (delay in days)**	**Partial response (delay in days)**	**Total dose to complete response (g)**	**Sodium thiosulfate initiation delay (days)**	**Side effects**	**Adjunctive treatments and interventions**
1	Yes (NA)	Yes (9)	450	27	Nausea-vomiting, dyspnea, volume overload	Non-calcium chelator, ↑ hemodialysis frequency and duration
2	Yes (115)	Yes (1)	1700	123	Nausea-vomiting, anorexia, weight loss, hypoglycemia	Bisphosphonate, cinacalcet, ↑ hemodialysis frequency
3	Yes (225)	Yes (30)	1650	47	Not documented	None
4	No	Yes (23)	NA	107	Volume overload	Bisphosphonate, ↑ hemodialysis frequency and duration, ↓ calcium dialysate
5	Yes (105)	Yes (NA)	750	13	None	Bisphosphonate
6	No	Yes (4)	NA	273	Nausea-vomiting	Bisphosphonate, cinacalcet, ↑ hemodialysis frequency, ↓ calcium dialysate
7	NA^a^	Yes (1)	NA^a^	1	Nausea-vomiting, headache, mild hypernatremia	None
8	NA^a^	NA	NA^a^	8	Nausea-vomiting	Non-calcium chelator, bisphosphonate,↓ calcium dialysate
9	Yes (133)	Yes (3)	1095	14	Volume overload, metabolic acidosis	Non-calcium chelator, bisphosphonate, ↑ hemodialysis frequency and duration, ↓ calcium dialysate
10	NA^a^	NA	NA^a^	40	Not documented	↓ calcium dialysate
11	No	No	NA	13	None	Bisphosphonate, cinacalcet
12	No	Yes (7)	NA	4	Nausea-vomiting	Non-calcium chelator, bisphosphonate, ↓ calcium dialysate

NA: Not applicable or not available; ↑: Increase; ↓: Decrease

^a^ Sodium thiosulfate stopped for side effects.

**Table 4.** No factor was associated with a complete response to sodium thiosulfate treatment.

Patients profile and interventions	Partial response (*n* = 4)	Complete response (*n* = 5)	*p* Value
Age (Mean ± SD, years)	62.50 ± 8.65	62.20 ± 8.82	0.97
Body mass index (Mean ± SD, kg/m^2^)	37.80 ± 5.71	33.14 ± 8.85	0.45
Obesity n (%)	3 (75)	3 (60)	1.00
Female n (%)	4 (100)	2 (40)	0.17
Bisphosphonate addition n (%)	3 (75)	3 (60)	1.00
↓ Calcium dialysate (Mean difference ± SE, mmol/L)	−0.07 ± 0.06	−0.16 ± 0.23	0.56
↑ Dialysis frequency (Mean ± SD, per week)	5.0 ± 2.3	3.0 ± 0	0.17
↑ Dialysis duration (Mean ± SD, hours)	3.50 ± 0.71	5.00 ± 2.04	0.39
Post-diagnosis sodium thiosulfate initiation delay (Mean ± SD, days)	96.25 ± 110.61	44.80 ± 40.98	0.42
Sodium thiosulfate treatment duration (Mean ± SD, days)	80.75 ± 46.64	102.20 ± 36.06	0.51

SE: Standard error; SD: standard deviation.

On the other hand, Spearman rank correlation test revealed no correlation between the total dose of STS and any of these three parameters: Post-diagnosis STS initiation delay, BMI and age (date not shown). Although the data we have within our cohort do not allow us to suggest standard dosages/frequencies for STS administration, we suggest reducing the dosage and/or frequency in case patients experience side effects and stop STS in case of major side effects.

### 3.5. Side effects of sodium thiosulfate treatment

The most frequently observed side effect was nausea and vomiting reported in six (50%) patients. It required antiemetic drugs in five (42%) patients and cessation of STS in 3 (25%) patients. During STS treatment, three patients developed a volume overload, resulting in diastolic heart failure for one of them. Only one patient presented a high anion gap metabolic acidosis that resolved following an adjustment of dialysate bicarbonate concentration.

### 3.6. Multimodality approach for calciphylaxis

In addition to STS, a multimodal approach was undertaken for 10 of our 12 patients with calciphylaxis (Table 3). Bisphosphonate (IV) was given to eight (68%) patients (Table 3 and Table 4), of which five patients received a single dose of 30, 60 or 90 mg and two patients received between three to five doses. Cinacalcet was started in three patients at a daily dosage of 30 mg after which one patient had the dose furtherly increased to 60 mg during the treatment period. At diagnosis, calcium-based phosphate binders were stopped in all patients and replaced with non-calcium-based phosphate binders. Active and non-active vitamin D were stopped in three and two patients respectively. Interestingly, nine (75%) patients were under warfarin for 3 ± 6 years when calciphylaxis was diagnosed. Due to the suggested link between warfarin and the development of vascular disease in CKD including calciphylaxis [3,40–42], warfarin was stopped in six patients and replaced by subcutaneous dalteparin. However, three patients continued warfarin as it has been judged necessary by their medical teams. In patients who were already on regular thrice weekly sessions of hemodialysis treatment, there was an intensification of the dialysis that varied from 1 supplementary hour per session to three supplementary dialysis sessions (total of 6 per week) in five patients. Moreover, dialysate calcium concentration was lowered from 1.25 to 1.00 mmol/L in 6 (50%) patients.

## 4. Discussion

In this retrospective study, we described the characteristics of twelve patients with CKD stage 5 diagnosed with calciphylaxis and treated with a multimodality approach including STS. We have shown that the majority of these patients presented risk factors suggested in the literature to be associated with calciphylaxis including diabetes, female gender, calcium-based phosphate binders therapy and active vitamin D administration. We report four key findings: 1) Treatment with STS combined to a multimodality approach successfully led to a complete or partial remission in 75% of our cohort; 2) No specific factor was associated with a complete therapeutic response to STS; 3) Warfarin seems to be associated with the development of calciphylaxis as 75% of our patients were under warfarin when calciphylaxis was diagnosed; 4) The one-year mortality in our cohort was lower than what is reported in the literature.

## 5. Key clinical features: Therapies, risks factors and mortality

STS is an inorganic compound that has been shown to inhibit medial arterial calcification in uremic rats (*in vivo*) and in cultured rat aortas (*in vitro*) through a potential direct extracellular action [43,44]. STS has also the ability to prevent the adipocyte induced arterial calcification (*in vitro*) [45]. However, the exact mechanisms of action of STS remain unclear and it was suggested that it does not probably involve calcium dissolution, anti-oxidant or pH effects as it was previously believed [46]. Hypotheses on the mechanisms of STS could involve the chelation [47] of calcium leading to calcium thiosulfate, a compound with an increased solubility [38,48] thus, reducing calcium mineral deposits [49]. The mechanism could also involve the anti-inflammatory properties of STS [50] that could contribute to both wound healing and pain relief.

As STS emerging role in calciphylaxis has been suggested, it is now used for the treatment of calciphylaxis worldwide especially in CKD patients [3,27,38,51–54]. Until now, there are no randomized studies that have evaluated the effects of STS in the treatment of calciphylaxis due to the rarity of this health problem and the difficulty to recruit a substantial number of patients. Therefore, the current available data in the literature are mainly based on case reports and from divergent experts opinions [10,46]. Recently, the working group on CKD-Mineral and Bone disorders from the European Renal Association-European Dialysis and Transplant Association (ERA-EDTA) has set up an European registry that will allow a better characterization (diagnosis, treatments and progression) of CKD patients diagnosed with calciphylaxis [7,55]. In our study, no correlation was found between the total dose and any of these three parameters: Post-diagnosis STS initiation delay, BMI and age (data not shown). This could indicate a certain efficacy of STS doses regardless of the post-diagnosis STS initiation delay, BMI and age; and further suggest that STS may be a good therapeutic choice for calciphylaxis as its actions would be independent from such clinical factors. Our analysis may also suggest a dose-dependent therapeutic effect of STS in patients who had a complete response. Since 2004, all CKD patients of our center diagnosed with calciphylaxis are treated with STS, starting at 25 g IV three times per week.

In addition to STS, our therapeutic approach is based on a multimodal treatment including bisphosphonates, cessation of warfarin, vitamin D and calcium-based phosphate binders, initiation of non-calcium-based phosphate binders and cinacalcet if needed, dialysis intensification and dialysate calcium concentration reduction until healing of the lesions. Parathyroidectomy is also considered for sever hyperparathyroidism cases that are unresponsive to medical treatment. In this study, if we consider both partial and complete responses, 75% of our patients had a positive therapeutic response. These results are similar or better than what was previously reported [56–59].

The average time before a complete response was 102.20 days (51–143 days) while the average STS treatment duration was 83.33 ± 41.52 days (median of 105 days). These data are in agreement with prior studies suggesting that at least 3 months of STS treatment was necessary before lesions improvement was observed [27,51,56]. The large interval of response delay observed in this study is probably due to the small number of patients, the different profiles of patients and the variability of the interventions for each patient. The delay before starting STS treatment also greatly varied within our cohort, which was probably due to limited STS availability at the pharmacy department when clinicians initially started this therapy. Although not statistically significant (probably due to the small number of patients in our cohort), an early STS treatment initiation in calciphylaxis has been advocated as an important contributing factor for treatment success [3,60]. In terms of side effects, 50% of STS treated patients experienced nausea or vomiting but symptoms were not severe enough to justify STS cessation. Globally, STS treatment was well tolerated by the majority of patients.

We also determined whether there were clinical or biological (laboratory) factors that were associated with a positive (either complete or partial) response to STS. On univariate analysis, no factor related to patients characteristics, comorbidities, lesion site, biochemical parameters or treatment modalities was identified to be associated with the healing or the non-healing of the lesions. However, such non-association might be the results of the limited statistical size of the studied group. Increasing the number of patients, whenever possible, would overcome such analysis limitations.

The clinical profiles of our patients are in agreement with previously published risk factors for calciphylaxis. Indeed, studies showed that diabetes, obesity, higher serum albumin and iPTH, use of cinacalcet [61], vitamin D and warfarin [61] were associated with an increased risk of developing calciphylaxis in a hemodialysis cohort population [62]. Herein, it is worth indicating that the association between calciphylaxis development risk and cinacalcet is probably only explained by an indication bias. Indeed, cinacalcet is prescribed for CKD patients, who already have an increased risk of developing calciphylaxis, for the treatment of hyperparathyroidism with the hope to reduce calciphylaxis-associated complications [63–66].

In contrast to the results of another recent study, we did not find any history of local trauma before diagnosis in our cohort [8]. Although our study was not meant to establish causality between warfarin use and calciphylaxis, we were not surprised to observe that the majority of our patients (*n* = 9) were under warfarin when diagnosed with calciphylaxis. Warfarin antagonizes vitamin K1 recycling, thus depleting active vitamin K1 and alters vitamin K metabolism [67]. Vitamin K is required for the activation of Matrix Gla protein, which is a known inhibitor of vascular calcification [68]. Vitamin K deficiency and treatment with warfarin may, therefore, predispose to the development of vascular calcification and calciphylaxis in CKD patients [41,62,69,70]. Thus, our results support that warfarin treatment should be used with extreme caution in advanced CKD population and especially those with known vascular calcification. Fortunately, oral anticoagulants represent alternative to warfarin as they do not interact with vitamin K 1 [71–73]. It is worth highlighting that the serum calcium, phosphorus and alkaline phosphatase levels of our patients were all within normal ranges which could indicate that abnormal mineral metabolism-related measures are not always associated to calciphylaxis. Finally, the heterogeneity of the side effects reflects different reactions of the patients depending on their pathological profiles as well as the preexisting comorbidities. This shows the complexity of establishing a therapeutic plan for such calciphylaxis patients who, in addition to CKD, have complex pathophysiological statuses.

The last important finding of this study is the low post-diagnosis one-year mortality rate. Calciphylaxis was historically associated with a high mortality rate (50–75%) at one year [56–58], whereas in this study it was 25%. The difference between our results and those from previous studies might be explained by the universal use of STS combined to a better wound care. Moreover, the use of a multimodality approach and the high level of awareness of diagnosis of calciphylaxis certainly improved the survival rate.

## 6. Study limitations, conclusion and perspectives

Interpreting these results requires to consider various elements, mainly the retrospective nature of this study, the absence of a control group and the limited size of the studied group. Due to the retrospective nature of this study, some data may have been missed during the data collection process, limiting some analysis. Furthermore, since the 12 clinical cases we reported were over a period of almost 8 years, both STS doses and the adjunctive treatments used in addition to STS were not standardized and have varied over time and were not identical for all patients. Thus, recommending an optimal dosage based on our study is not possible (no dose-dependant studies). However, reducing the doses (we typically decrease by 50%) in case of side effects could be a recommended approach. Since 2004, STS was systematically added in the treatment protocol of calciphylaxis at our center. Unlike the usual pharmacological clinical studies, our study did not involve a control group. To improve the design, our analyses were expanded to compare various parameters and calculate correlation parameters. Importantly, comparing patients with a complete response to those who had only a partial response would partially overcome the fact of not having a control group. This can even have the advantage of comparing two groups in which the drug shows effects.

CKD patients who develop calciphylaxis often suffer from multiple comorbidities and are frequently treated with warfarin. However, no factors were shown to be associated with a complete therapeutic response to STS. It is worth precising that the limitation of our study could make that the statistical conclusion can only be taken as indications that are still important. Indeed, although our sample size might have limited statistical power compared to the standards in clinical studies, the rarity of calciphylaxis cases makes our results an important addition to the available clinical data. Moreover, for numerous data and observations, even though they were not a part of the analyses and comparisons we performed, we still provided them under the results section to enrich the available literature on calciphylaxis.

As calciphylaxis represents a rare complication, collecting the reported clinical cases, along with the prescribed therapies and the corresponding responses, will allow to establish international registries. Such registries will record the reported calciphylaxis cases worldwide, the patients’ profiles, STS therapeutic regimen, adjunctive therapies, prognosis, side effects, etc. This will lead to the optimization of calciphylaxis therapies based on the compilation of all the reported information similarly to the Australian Calciphylaxis Registry [74], the registry of the international society for heart and lung transplantation [75,76], the international severe asthma registry [77], the world apheresis association registry [78] and the international takotsubo registry [79]. Such registries can overcome the statistical limitations and provide strong data to orient the clinicians to the most optimized therapeutic approaches based on the patients profiles and prognosis. Finally, with the newer expanded dialytic therapies like hemodiafiltration [80] or the expanded hemodialysis with medium cutoff membranes [81] dialyzers, we expect an even better treatment outcomes for calciphylaxis [82].

## References

[1] Zhou Y, Chen Y, Yin G, et al. Calciphylaxis and its co-occurrence with connective tissue diseases. Int Wound J. 2023;20(4):1–10. doi: 10.1111/iwj.13972. PMC10031236 36274216

[2] Gallo Marin B, Aghagoli G, Hu SL, et al. Calciphylaxis and kidney disease: a review. Am J Kidney Dis. 2023;81(2):232–239. doi: 10.1053/j.ajkd.2022.06.011. 35970430

[3] Nigwekar SU, Kroshinsky D, Nazarian RM, et al. Calciphylaxis: risk factors, diagnosis, and treatment. Am J Kidney Dis. 2015;66(1):133–146. doi: 10.1053/j.ajkd.2015.01.034. 25960299 PMC4696752

[4] Kaur R, Singh R. Mechanistic insights into CKD-MBD-related vascular calcification and its clinical implications. Life Sci. 2022;311(Pt B):121148. doi: 10.1016/j.lfs.2022.121148. 36336124

[5] Brandenburg VM, Kramann R, Specht P, et al. Calciphylaxis in CKD and beyond. Nephrol Dial Transplant. 2012;27(4):1314–1318. doi: 10.1093/ndt/gfs015. 22344774

[6] Fine A, Zacharias J. Calciphylaxis is usually non-ulcerating: risk factors, outcome and therapy. Kidney Int. 2002;61(6):2210–2217. doi: 10.1046/j.1523-1755.2002.00375.x. 12028462

[7] Brandenburg VM, Kramann R, Rothe H, et al. Calcific uraemic arteriolopathy (calciphylaxis): data from a large nationwide registry. Nephrol Dial Transplant. 2017;32(1):126–132. doi: 10.1093/ndt/gfv438. 26908770

[8] Nigwekar SU, Zhao S, Wenger J, et al. A nationally representative study of calcific uremic arteriolopathy risk factors. J Am Soc Nephrol. 2016;27(11):3421–3429. doi: 10.1681/ASN.2015091065. 27080977 PMC5084892

[9] Chang JJ. Calciphylaxis: diagnosis, pathogenesis, and treatment. Adv Skin Wound Care. 2019;32(5):205–215. doi: 10.1097/01.ASW.0000554443.14002.13. 31008757

[10] Brandenburg VM, Cozzolino M, Ketteler M. Calciphylaxis: a still unmet challenge. J Nephrol. 2011;24(2):142–148. doi: 10.5301/jn.2011.6366. 21337312

[11] Toussaint ND, Lau KK, Strauss BJ, et al. Associations between vascular calcification, arterial stiffness and bone mineral density in chronic kidney disease. Nephrol Dial Transplant. 2008;23(2):586–593. doi: 10.1093/ndt/gfm660. 17933842

[12] Rick J, Strowd L, Pasieka HB, et al. Calciphylaxis: part I. Diagnosis and pathology. J Am Acad Dermatol. 2022;86(5):973–982. doi: 10.1016/j.jaad.2021.10.064. 35114300

[13] Jiao Y, Sun L, Xie X, et al. Clinical features and outcomes of calciphylaxis in chinese patients with chronic kidney disease. Nephrology (Carlton). 2023;28(6):305–314. doi: 10.1111/nep.14156. 36883928

[14] Weenig RH, Sewell LD, Davis MD, et al. Calciphylaxis: natural history, risk factor analysis, and outcome. J Am Acad Dermatol. 2007;56(4):569–579. doi: 10.1016/j.jaad.2006.08.065. 17141359

[15] Xia J, Tan AJ, Biglione B, et al. Nephrogenic calciphylaxis arising after bariatric surgery: a case series. Am J Nephrol. 2023. Online ahead of print. doi: 10.1159/000531784. 37487472

[16] Farah M, Crawford RI, Levin A, et al. Calciphylaxis in the current era: emerging ‘ironic’ features? Nephrol Dial Transplant. 2011;26(1):191–195. doi: 10.1093/ndt/gfq407. 20627865

[17] Jean G, Terrat JC, Vanel T, et al. [Calciphylaxis in dialysis patients: to recognize and treat it as soon as possible]. Nephrol Ther. 2010;6(6):499–504. doi: 10.1016/j.nephro.2010.04.003. 20627839

[18] Wang HY, Yu CC, Huang CC. Successful treatment of severe calciphylaxis in a hemodialysis patient using low-calcium dialysate and medical parathyroidectomy: case report and literature review. Ren Fail. 2004;26(1):77–82. doi: 10.1081/jdi-120028559. 15083927

[19] Smith R, Bulteel N, Gupta AA. S. Successful treatment of severe calciphylaxis in a renal transplant patient with previous total parathyroidectomy. J R Coll Physicians Edinb. 2023; 10.1177/14782715231184519 37427771

[20] Sinha S, Gould LJ, Nigwekar SU, et al. The CALCIPHYX study: a randomized, double-blind, placebo-controlled, phase 3 clinical trial of SNF472 for the treatment of calciphylaxis. Clin Kidney J. 2022;15(1):136–144. doi: 10.1093/ckj/sfab117. 35035944 PMC8757410

[21] Perelló J, Ferrer MD, del Mar Pérez M, et al. Mechanism of action of SNF472, a novel calcification inhibitor to treat vascular calcification and calciphylaxis. Br J Pharmacol. 2020;177(19):4400–4415. doi: 10.1111/bph.15163. 32557649 PMC7484563

[22] Bian A, Ye X, Wang J, et al. Therapeutic effects and mechanism of human amnion-derived mesenchymal stem cells on hypercoagulability in a uremic calciphylaxis patient. Ren Fail. 2023;45(1):2218483. doi: 10.1080/0886022X.2023.2218483. 37293809 PMC10259294

[23] Qin L, Zhang J, Xiao Y, et al. A novel long-term intravenous combined with local treatment with human amnion-derived mesenchymal stem cells for a multidisciplinary rescued uremic calciphylaxis patient and the underlying mechanism. J Mol Cell Biol. 2022;14(2):mjac010. doi: 10.1093/jmcb/mjac010. 35142858 PMC9205756

[24] Wang NN, Qin LJ, Liu K[, et al. Multidisciplinary regenerative treatment and mechanisms for rescuing a severe calciphylaxis patient with human amnion-derived mesenchymal stem cells. ]. Zhonghua Yi Xue Za Zhi. 2022;102(28):2217–2221. 35872588 10.3760/cma.j.cn112137-20211218-02819

[25] Kelly E, O’Hagan J. Geographic clustering of economic activity: the case of prominent Western visual artists. J Cult Econ. 2007;31:109–128. doi: 10.1007/s10824-007-9035-x.

[26] Malbos S, Urena-Torres P, Bardin T, et al. Sodium thiosulfate is effective in calcific uremic arteriolopathy complicating chronic hemodialysis. Joint Bone Spine. 2016;83(1):89–92. doi: 10.1016/j.jbspin.2015.03.007. 26494591

[27] Hayden MR, Tyagi SC, Kolb L, et al. Vascular ossification-calcification in metabolic syndrome, type 2 diabetes mellitus, chronic kidney disease, and calciphylaxis-calcific uremic arteriolopathy: the emerging role of sodium thiosulfate. Cardiovasc Diabetol. 2005;4(1):4. doi: 10.1186/1475-2840-4-4. 15777477 PMC1079905

[28] Araya CE, Fennell RS, Neiberger RE, et al. Sodium thiosulfate treatment for calcific uremic arteriolopathy in children and young adults. Clin J Am Soc Nephrol. 2006;1(6):1161–1166. doi: 10.2215/CJN.01520506. 17699342

[29] Di J, Liu Y, Wang D, et al. A case of early calciphylaxis diagnosed by bone scan. Case Rep Med. 2020;2020:9526836. 32256605 10.1155/2020/9526836 PMC7103050

[30] Paul S, Rabito CA, Vedak P, et al. The role of bone scintigraphy in the diagnosis of calciphylaxis. JAMA Dermatol. 2017;153(1):101–103. doi: 10.1001/jamadermatol.2015.4591. 26677101

[31] Han MM, Pang J, Shinkai K, et al. Calciphylaxis and bone scintigraphy: case report with histological confirmation and review of the literature. Ann Nucl Med. 2007;21(4):235–238. doi: 10.1007/s12149-007-0013-3. 17581723

[32] Ghosh T, Winchester DS, Davis MDP, et al. Early clinical presentations and progression of calciphylaxis. Int J Dermatol. 2017;56(8):856–861. doi: 10.1111/ijd.13622. 28436018

[33] Bliss DE. Calciphylaxis: what nurses need to know. Nephrol Nurs J. 2002;29(5):433–438. 443-434; quiz 445–436. 12434450

[34] Khanna U, Dominguez A, Keller J, et al. Update on calciphylaxis etiopathogenesis, diagnosis, and management. Cutis. 2018;102(6):395–400. 30657805

[35] Zoi V, Bacharaki D, Sardeli A, et al. Calciphylaxis: a long road to cure with a multidisciplinary and multimodal approach. Case Rep Nephrol. 2022;2022:3818980. doi: 10.1155/2022/3818980. 35720957 PMC9200596

[36] Yang X, Liu Y, Xie X, et al. Use of the optimized sodium thiosulfate regimen for the treatment of calciphylaxis in chinese patients. Ren Fail. 2022;44(1):914–922. doi: 10.1080/0886022X.2022.2081179. 35634730 PMC9154757

[37] Brandenburg VM, Cozzolino M, Mazzaferro S. Calcific uremic arteriolopathy: a call for action. Semin Nephrol. 2014;34(6):641–647. doi: 10.1016/j.semnephrol.2014.09.007. 25498382

[38] Schlieper G, Brandenburg V, Ketteler M, et al. Sodium thiosulfate in the treatment of calcific uremic arteriolopathy. Nat Rev Nephrol. 2009;5(9):539–543. doi: 10.1038/nrneph.2009.99. 19701230

[39] Brandenburg VM, Evenepoel P, Floege J, et al. Lack of evidence does not justify neglect: how can we address unmet medical needs in calciphylaxis? Nephrol Dial Transplant. 2016;31(8):1211–1219. doi: 10.1093/ndt/gfw025. 27005994

[40] Al-Ani M, Parperis K. Warfarin-induced calciphylaxis. BMJ Case Rep. 2016;2016:bcr2015214142. 10.1136/bcr-2015-214142 PMC4735329 26783010

[41] Mac-Way F, Poulin A, Utescu MS, et al. The impact of warfarin on the rate of progression of aortic stiffness in hemodialysis patients: a longitudinal study. Nephrol Dial Transplant. 2014;29(11):2113–2120. doi: 10.1093/ndt/gfu224. 24944209

[42] Santos PW, Wetmore JB. Sequential bone scintigraphy and the evolution of Warfarin-Mediated calcific uremic arteriolopathy. Case Rep Nephrol Dial. 2021;11(1):78–86. doi: 10.1159/000512611. 33829045 PMC7991460

[43] O’Neill WC, Hardcastle KI. The chemistry of thiosulfate and vascular calcification. Nephrol Dial Transplant. 2012;27(2):521–526. doi: 10.1093/ndt/gfr375. 21737516 PMC3275785

[44] Pasch A, Schaffner T, Huynh-Do U, et al. Sodium thiosulfate prevents vascular calcifications in uremic rats. Kidney Int. 2008;74(11):1444–1453. doi: 10.1038/ki.2008.455. 18818688

[45] Chen NX, O’Neill K, Akl NK, et al. Adipocyte induced arterial calcification is prevented with sodium thiosulfate. Biochem Biophys Res Commun. 2014;449(1):151–156. doi: 10.1016/j.bbrc.2014.05.005. 24824185

[46] O’Neill WC. Sodium thiosulfate: mythical treatment for a mysterious disease? Clin J Am Soc Nephrol. 2013;8(7):1068–1069. 23743999 10.2215/CJN.04990513

[47] Adirekkiat S, Sumethkul V, Ingsathit A, et al. Sodium thiosulfate delays the progression of coronary artery calcification in haemodialysis patients. Nephrol Dial Transplant. 2010;25(6):1923–1929. doi: 10.1093/ndt/gfp755. 20083471

[48] Generali JA, Cada DJ. Sodium thiosulfate: calciphylaxis. Hosp Pharm. 2015;50(11):975–977. doi: 10.1310/hpj5011-975. 27621504 PMC4750847

[49] Kreymereman P. Sodium thiosulfate injection dissolves calcium hydroxylapatite particles: an animal study. Journal of the American Academy of Dermatology. 2018;79(3, Supplement 1):AB265.

[50] Zhang MY, Dugbartey GJ, Juriasingani S, et al. Hydrogen sulfide metabolite, sodium thiosulfate: clinical applications and underlying molecular mechanisms. Int J Mol Sci. 2021;22(12):6452. 34208631 10.3390/ijms22126452 PMC8235480

[51] Hayden MR, Goldsmith DJ. Sodium thiosulfate: new hope for the treatment of calciphylaxis. Semin Dial. 2010;23(3):258–262. doi: 10.1111/j.1525-139X.2010.00738.x. 20636917

[52] Wen W, Portales-Castillo I, Seethapathy R, et al. Intravenous sodium thiosulphate for calciphylaxis of chronic kidney disease: a systematic review and meta-analysis. JAMA Netw Open. 2023;6(4):e2310068. doi: 10.1001/jamanetworkopen.2023.10068. 37099293 PMC10134003

[53] Peng T, Zhuo L, Wang Y, et al. Systematic review of ­sodium thiosulfate in treating calciphylaxis in chronic kidney disease patients. Nephrology (Carlton). 2018;23(7):669–675. doi: 10.1111/nep.13081. 28603903

[54] Teh YK, Renaud CJ. Clinical experience with intraperitoneal sodium thiosulphate for calciphylaxis in peritoneal dialysis: a case series. Perit Dial Int. 2023; doi: 10.1177/08968608231163669. 37131321

[55] Brandenburg V, Adragao T, van Dam B, et al. Blueprint for a european calciphylaxis registry initiative: the european calciphylaxis network (EuCalNet). Clin Kidney J. 2015;8(5):567–571. doi: 10.1093/ckj/sfv056. 26413282 PMC4581376

[56] Sood AR, Wazny LD, Raymond CB, et al. Sodium thiosulfate-based treatment in calcific uremic arteriolopathy: a consecutive case series. Clin Nephrol. 2011;75(1):8–15. 21176746

[57] Noureddine L, Landis M, Patel N, et al. Efficacy of sodium thiosulfate for the treatment for calciphylaxis. Clin Nephrol. 2011;75(6):485–490. doi: 10.5414/cnp75485. 21612750

[58] Zitt E, Konig M, Vychytil A, et al. Use of sodium thiosulphate in a multi-interventional setting for the treatment of calciphylaxis in dialysis patients. *Nephrol Dial Transplant*. 2013. 10.1093/ndt/gfs548 23291368

[59] Bourgeois P, Haes DP . Sodium thiosulfate as a treatment for calciphylaxis: a case series. J Dermatolog Treat. 2016;27(6):520–524. 27051974 10.3109/09546634.2016.1163316

[60] Loidi Pascual L, Valcayo Penalba A, Oscoz Jaime S, et al. [Calciphylaxis. A review of 9 cases]. Med Clin (Barc). 2016;147(4):157–161. doi: 10.1016/j.medcli.2016.05.021. 27422736

[61] Jang A, Mark Ho MH, Yan L. Characterization of risk factors for calciphylaxis in hemodialysis patients in the fraser health renal Program - A matched Case-Control retrospective review. J Pharm Pract. 2022; 10.1177/08971900221118444 36036088

[62] Jovanovich A, Chonchol M. Calcific uremic arteriolopathy revisited. J Am Soc Nephrol. 2016;27(11):3233–3235. doi: 10.1681/ASN.2016040480. 27225039 PMC5084902

[63] Sun Y, Tian B, Sheng Z, et al. Efficacy and safety of cinacalcet compared with other treatments for secondary hyperparathyroidism in patients with chronic kidney disease or end-stage renal disease: a meta-analysis. BMC Nephrol. 2020;21(1):316. doi: 10.1186/s12882-019-1639-9. 32736534 PMC7393724

[64] Xu J, Yang Y, Ma L, et al. Cinacalcet plus vitamin D versus vitamin D alone for the treatment of secondary hyperparathyroidism in patients undergoing dialysis: a meta-analysis of randomized controlled trials. Int Urol Nephrol. 2019;51(11):2027–2036. doi: 10.1007/s11255-019-02271-6. 31531805

[65] Deen J, Schaider H. The use of cinacalcet for the treatment of calciphylaxis in patients with chronic kidney disease: a comprehensive review. Australas J Dermatol. 2019;60(3):e186–e194. doi: 10.1111/ajd.12992. 30666627

[66] Raymond CB, Wazny LD. Sodium thiosulfate, bisphosphonates, and cinacalcet for treatment of calciphylaxis. Am J Health Syst Pharm. 2008;65(15):1419–1429. doi: 10.2146/ajhp070546. 18653812

[67] Rishavy MA, Hallgren KW, Wilson L, et al. Warfarin alters vitamin K metabolism: a surprising mechanism of VKORC1 uncoupling necessitates an additional reductase. Blood. 2018;131(25):2826–2835. doi: 10.1182/blood-2017-09-804666. 29592891 PMC6014353

[68] Brandenburg VM, Schurgers LJ, Kaesler N, et al. Prevention of vasculopathy by vitamin K supplementation: can we turn fiction into fact? Atherosclerosis. 2015;240(1):10–16. doi: 10.1016/j.atherosclerosis.2015.02.040. 25744701

[69] Jean G, Bresson E, Terrat JC, et al. Peripheral vascular calcification in long-haemodialysis patients: associated factors and survival consequences. Nephrol Dial Transplant. 2009;24(3):948–955. doi: 10.1093/ndt/gfn571. 18852190

[70] Caluwe R, Pyfferoen L, De Boeck K, et al. The effects of vitamin K supplementation and vitamin K antagonists on progression of vascular calcification: ongoing randomized controlled trials. Clin Kidney J. 2016;9(2):273–279. doi: 10.1093/ckj/sfv146. 26985380 PMC4792621

[71] Khairani CD, Bejjani A, Piazza G, et al. Direct oral anticoagulants vs vitamin K antagonists in patients with antiphospholipid syndromes: meta-Analysis of randomized trials. J Am Coll Cardiol. 2023;81(1):16–30. doi: 10.1016/j.jacc.2022.10.008. 36328154 PMC9812926

[72] Dufrost V, Wahl D, Zuily S. Direct oral anticoagulants in antiphospholipid syndrome: meta-analysis of randomized controlled trials. Autoimmun Rev. 2021;20(1):102711. doi: 10.1016/j.autrev.2020.102711. 33197580

[73] Wu X, Cao S, Yu B, et al. Comparing the efficacy and safety of direct oral anticoagulants versus vitamin K antagonists in patients with antiphospholipid syndrome: a systematic review and meta-analysis. Blood Coagul Fibrinolysis. 2022;33(7):389–401. doi: 10.1097/MBC.0000000000001153. 35867933 PMC9594143

[74] Ruderman I, Toussaint ND, Hawley CM, et al. The Australian calciphylaxis registry: reporting clinical features and outcomes of patients with calciphylaxis. Nephrol Dial Transplant. 2021;36(4):649–656. doi: 10.1093/ndt/gfz256. 31855262

[75] Boucek MM, Aurora P, Edwards LB, et al. Registry of the international society for heart and lung transplantation: tenth official pediatric heart transplantation report–2007. J Heart Lung Transplant. 2007;26(8):796–807. doi: 10.1016/j.healun.2007.06.006. 17692783

[76] Stehlik J, Edwards LB, Rowe A, et al. ISHLT international registry for heart and lung transplantation - three decades of scientific contributions. Transplant Rev (Orlando). 2013;27(2):38–42. doi: 10.1016/j.trre.2013.01.005. 23465193

[77] International severe asthma registry: mission statement. Chest. 2020;157(4):805–814. 31838187 10.1016/j.chest.2019.10.051

[78] Rock G. International forum: the world apheresis association registry. Transfus Apher Sci. 2023;62(1):103630. doi: 10.1016/j.transci.2022.103630. 36639290

[79] Ghadri JR, Cammann VL, Templin C. The international takotsubo registry: rationale, design, objectives, and first results. Heart Fail Clin. 2016;12(4):597–603. doi: 10.1016/j.hfc.2016.06.010. 27638029

[80] Golper TA. Hemodiafiltration outcomes in special situations. Semin Dial. 2022;35(5):431–435. doi: 10.1111/sdi.13074. 35315945

[81] Zhang Z, Yang T, Li Y, et al. Effects of expanded hemodialysis with medium Cut-Off membranes on maintenance hemodialysis patients: a review. Membranes. 2022;12(3):253. doi: 10.3390/membranes12030253. 35323729 PMC8953230

[82] Valga F, Monzón T, Rincón M, et al. Synergy of sodium thiosulphate treatment and expanded hemodialysis in the management of calciphylaxis? A case report. Nefrologia (Engl Ed). 2022;42(3):354–356. doi: 10.1016/j.nefroe.2022.09.001. 36153291

